# Detection of *Pseudomonas aeruginosa* Serogroup G Using Real-Time PCR for Novel Target Genes Identified Through Comparative Genomics

**DOI:** 10.3389/fmicb.2022.928154

**Published:** 2022-06-24

**Authors:** Chufang Wang, Qinghua Ye, Yu Ding, Jumei Zhang, Qihui Gu, Rui Pang, Hui Zhao, Juan Wang, Qingping Wu

**Affiliations:** ^1^College of Food Science, South China Agricultural University, Guangzhou, China; ^2^Guangdong Provincial Key Laboratory of Microbial Safety and Health, State Key Laboratory of Applied Microbiology Southern China, Institute of Microbiology, Guangdong Academy of Sciences, Guangzhou, China; ^3^Key Laboratory of Agricultural Microbiomics and Precision Application, Ministry of Agriculture and Rural Affairs, Guangdong Academy of Sciences, Guangzhou, China

**Keywords:** serogroup G-specific target, aquatic environment, molecular detection, serotyping, sensitivity

## Abstract

Accurate serotyping is essential for effective infection control. *Pseudomonas aeruginosa* serogroup G is one of the most common serogroups found in water. Conventional serotyping methods are not standardized and have several shortcomings. Therefore, a robust method for rapidly identifying *P. aeruginosa* serotypes is required. This study established a real-time PCR method for identifying *P. aeruginosa* serogroup G strains using novel target gene primers based on comparative genomic analysis. A total of 343 genome sequences, including 16 *P. aeruginosa* serogroups and 67 other species, were analyzed. Target genes identified were amplified using real-time PCR for detecting *P. aeruginosa* serogroup G strains. Eight serogroup G genes, *PA59_01276, PA59_01887, PA59_01888, PA59_01891, PA59_01894, PA59_04268, PA59_01892*, and *PA59_01896*, were analyzed to determine specific targets. A real-time fluorescence quantitative PCR method, based on the novel target *PA59_01276*, was established to detect and identify serogroup G strains. The specificity of this method was confirmed using *P. aeruginosa* serogroups and non-*P. aeruginosa* species. The sensitivity of this real-time PCR method was 4 × 10^2^ CFU/mL, and it could differentiate and detect *P. aeruginosa* serogroup G in the range of 4.0 × 10^3^–4.0 × 10^8^ CFU/mL in artificially contaminated drinking water samples without enrichment. The sensitivity of these detection limits was higher by 1–3 folds compared to that of the previously reported PCR methods. In addition, the G serum group was accurately detected using this real-time PCR method without interference by high concentrations of artificially contaminated serum groups F and D. These results indicate that this method has high sensitivity and accuracy and is promising for identifying and rapidly detecting *P. aeruginosa* serogroup G in water samples. Moreover, this research will contribute to the development of effective vaccines and therapies for infections caused by multidrug-resistant *P. aeruginosa*.

## Introduction

*Pseudomonas aeruginosa* (*P. aeruginosa*) is a widespread pathogen found in water. It is a versatile opportunistic pathogen that thrives in moist environments, such as soil and water, causing water-borne diseases and nosocomial infections (Falkinham et al., 2015). In recent decades, the number of *P. aeruginosa-*related water-borne illnesses has increased dramatically (Catho et al., 2021; Petitjean et al., 2021). *P. aeruginosa* infection may cause a variety of diseases, including those of the respiratory tract (predominantly cystic fibrosis), circulatory system (bacteremia and sepsis), central nervous system, heart (endocarditis), ears (including otitis external), eyes, bones, gastrointestinal tract, urinary tract, and skin (Carmeli et al., 2016; Lee et al., 2017; Morand and Morand, 2017). The epidemiology, virulence, and drug resistance of *P. aeruginosa* are closely related to its serotype (Ayi, 2015; Catho et al., 2021).

Serotyping is a phenotypic typing technique used for epidemiological sorting, clinical drug resistance analysis, disease transmission monitoring, and *P. aeruginosa* infection source tracing (Thrane et al., 2016; Del Barrio-Tofino et al., 2019). Based on the O-specific antigen (OSA, B-band), *P. aeruginosa* is classified into 14 serogroups (serogroups A-N), which correspond to the early international typing of O antigen serum (O1–O20) (Homma, 1982; Knirel et al., 2010; Lam et al., 2011; Zhao et al., 2018). There is no difference between the serogroups G and O6 in the reaction principle, classification, and scope of inclusion, except for the name (Riaz and Hashmi, 2019; Nasrin et al., 2022). Serogroup G, often present in food and water, is the most widespread serotype, and one of the five most frequently studied serotypes (Faure et al., 2003; Mena and Gerba, 2009; Koch et al., 2014; Ayi, 2015; Balabanova et al., 2020; Howlader et al., 2021). Serogroup G is considered the primary cause of burn wound infections, and it is one of the predominant *P. aeruginosa* serotypes among clinical isolates (Pirnay et al., 2002). A link exists between the clinical prevalence and O antigen serotype, such as among 1445 *P. aeruginosa* isolates analyzed from humans, 17.8% were identified as serogroup G (Qi et al., 2014; Del Barrio-Tofino et al., 2019). The *P. aeruginosa* serogroup G strain ST3449 shows resistance to multiple antibiotics and often leads to lung diseases such as cystic fibrosis and multiple drug resistant in infection in patients (Diaz-Rios et al., 2021). *P. aeruginosa* serogroup G is exceptionally resistant to antibiotics (over 90%), leading to a high mortality rate in burn victims (Nasrin et al., 2022). Rapid identification of the serogroup G strain is essential for early diagnosis.

Routine detection of *P. aeruginosa* serotypes is based on biochemical and slide agglutination tests. Determining the serotype of *P. aeruginosa* requires 5–8 days. In addition, slide agglutination has several drawbacks: it is time-consuming, labor-intensive, requires high-quality antiserum, and requires standardization of the antiserum (Koch et al., 2014; Allison and Castric, 2016; Thrane et al., 2016; Li et al., 2018). There are similarities between the different *P. aeruginosa* serotyping systems; however, the reason for the choice which serotyping method is not apparent. The conventional serotyping methods are not standard, and there are at least eight serotyping methods for *P. aeruginosa*. In addition, existing serotyping plans do not cover all serotypes of *P. aeruginosa* (Kaluzny et al., 2007; Kintz et al., 2008; Koch et al., 2014). A serotype kit for *P. aeruginosa* strains evaluated in which 37.5% of the strains were non-typeable (Le Berre et al., 2011). Serotyping is the most common approach for identifying *P. aeruginosa* strain; however, molecular typing and identification methods must be incorporated into epidemiological research for determining the relationship between the disease and the source of *P. aeruginosa* contamination.

Polymerase chain reaction is favored for pathogen detection because of its specificity, sensitivity, rapidity, and simplicity. Real-time PCR often used to ascertain product safety, quality, and authenticity. Several methods related to serum detection have also been reported, such as Real-time PCR method, which permits continuous monitoring of reaction progress, quantification of target DNA (Koch et al., 2014; Li et al., 2018). Nevertheless, these studies are restricted by incomplete strain information or the unavailability of a figure from an available public genomics library, resulting in a lack of detection targets. Therefore, identifying other OSA-related genes in the *P. aeruginosa* genome could be beneficial (Thrane et al., 2016).

**FIGURE 1:**
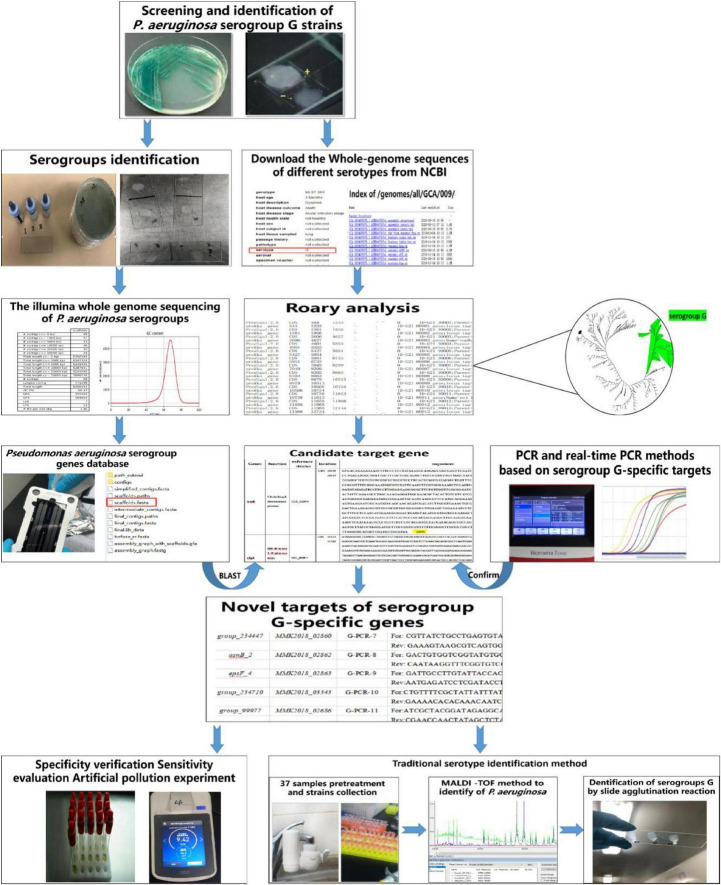
The flowchart of the experimental method involved in this study.

The evolution of bioinformatics and whole-genome sequencing technology has enabled obtaining the genome sequence of *P. aeruginosa* with serogroup information; the sequences can be obtained from the National Biotechnology Information Center (NCBI^1^). Considering the research on vaccine efficacy and drug development and the shortcomings of traditional serotyping methods, a robust molecular typing method for the specific identification of *P. aeruginosa* serogroup G is essential. Therefore, we aimed to use comparative genomic analysis to identify new molecular targets for different *P. aeruginosa* serotypes and determine new serogroup G-specific molecular targets to establish a sensitive real-time PCR method for the rapid quantitative detection of *P. aeruginosa* serogroup G. Furthermore, the established methods were applied to the detection of actual water samples to provide a faster and efficient ways for the risk investigation of water pollution and provide a scientific basis for reducing pollution. The flowchart of the experimental method involved in this study is shown in Figure 1.

## Materials and Methods

### Mining of *P. aeruginosa* Serogroup G-Specific Targets

A total of 343 genome sequences, including those of 16 *P. aeruginosa* serotypes and 67 other species, were downloaded from the NCBI Genome database (see text footnote 1). Bacterial whole-genome sequences contain informative features of their evolutionary pathways, and accurately discriminate among populations, strains or closely related species. In this study, in order to ensure that the obtained target genes have excellent specificity, the sequences of selected genes need higher coverage and highly homologous with other genes. Of the 67 species used for analysis, 54 different species belong to the same *pseudomonas* genus, the remaining species are gram – negative and gram – positive representative species. For *P. aeruginosa*, 12,439 genomes were uploaded to NCBI, of which 142 genomes (contigs ≤ 200) carried serum-related information. Information regarding the *Pseudomonas* genomes is presented in Supplementary Table 1. Genome annotation was performed on all analyzed isolates using Prokka v1.11 (Seemann, 2014). The Prokka output was used to construct the pan-genome using Roary v3.11.2 (Page et al., 2015). A core genome was determined for each isolate using a 99% cutoff with a BLASTP identity cutoff of 85% (Pang et al., 2019). Genes that matched all *P. aeruginosa* serogroup G genomic sequences were considered highly conserved. They were used in the subsequent alignment of genomic sequences from other *P. aeruginosa* serotypes and other *Pseudomonas* species. Using Harvest v1.1.2 to generate the core genome alignment of *P. aeruginosa* with the ATCC33350 genome as a reference (Treangen et al., 2014). After remove the putative recombined regions, Genealogies Unbiased by recomBinations In Nucleotide Sequences (Gubbins) was used for recombination analysis (Li et al., 2022). Single nucleotide polymorphisms (SNPs) were extracted from the recombination-free core-genome alignment using the script available at https://github.com/sanger-pathogens/snp-sites. Based on the SNP alignment, FastTree v.2.1.10 with the general time-reversible (GTR) and gamma model of nucleotide substitution were used to construct a maximum-likelihood (ML) phylogenetic tree (Cheng et al., 2021). Using iTOL to visualize and annotate the ML phylogeny, and the results showed in Supplementary Figure 1 (Letunic and Bork, 2019). Specific genes were screened according to the following criteria: 100% presence in *P. aeruginosa* serogroup G strains and lack of presence in non-target *P. aeruginosa* strains. These candidate targets were then screened against the nucleotide collection (nr/nt) databases using the online BLAST program^2^ to ensure specificity.

### Bacterial Strains and Genomic DNA Extraction

In total, 254 strains were isolated from water (using the Chinese National Standard method with some modifications, GB 8537-2016) and food samples (using the standard SN/T 5228.9-2019 with some modifications), including 222 *P. aeruginosa* strains belonging to 13 serotypes and 24 non-*P. aeruginosa* strains were used (Table 1). All bacterial strains were cultured in Luria-Bertani (LB) broth at 37°C. Bacterial cultures were collected *via* centrifugation at 12,000 × *g* for 5 min. Genomic DNA from these cells was extracted and purified using ENZ and a Bacterial Genome Kit (Omega Bio-Tek Inc., Norcross, GA, United States), as per the manufacturer’s instructions. The concentration and purity of DNA were estimated using agarose gel electrophoresis and a NanoDrop 2000c UV- is spectrophotometer (Thermo Fisher Scientific, Waltham, MA, United States). The genomic DNA was stored at −20°C until use.

**TABLE 1:** Bacterial strains used in this study and PCR specificity results.

Bacterial species	Polyvalent serogroup	Monovalen serogroup (Number of strains)	Strains ID	*Source				PCR results								
					
									*PA59_01276*	*PA59_01887*	*PA59_01888*	*PA59_01891*	*PA59_01894*	*PA59_01892*	*PA59_04268*	*PA59_01896*
*Pseudomonas aeruginosa*	III	G (*n* = 18)	PA5	PA9	PA14	PA23	JR7-2	α, β, γ	+	+	+	+	+	+	+	+
			16C07	16C76	16C91	17C52	NR2-2									
			19C31	206038	206039	206052	206108									
			206091	206101	206104											
		D (*n* = 10)	15C06	16C106	16C79	16C78	XC4-2	α, γ	−	−	−	−	−	−	−	−
			15C28	16C59	19C28	16C01	XC4-2									
		E (*n* = 11)	15C05	16C53	17C44	17C105	NR1-3	α, γ	−	−	−	−	−	−	−	−
			15C17	16C80	17C67	17C96	NR1-3									
			16C40													
		F (*n* = 8)	15C16	16C100	16C95	SC4	206066	α, β, γ	−	−	−	−	−	−	−	−
			15C19	16C16	206037											
	I	A (*n* = 15)	16C04	16C18	16C31	16C02	NR1-1	α, γ	−	−	−	−	−	−	−	−
			16C107	16C19	16C36	17C64	SC3-1								−	−
			16C12	16C21	16C39	17C91	SC3-2									
		C (*n* = 8)	16C38	16C51	16C83		GIM1.46	α, γ	−	−	−	−	−	−	−	−
			16C46	16C62	17C73		HG4-2									
		H (*n* = 2)	206071	PA3				α, γ	−	−	−	−	−	−	−	−
		I (*n* = 23)	PA27	16C10	16C92	16C08	19C26	α, β, γ	−	−	−	−	−	−	−	−
			PA43	16C11	17C100	16C87	19C29									
			15C11	16C37	17C71	19C18	CMCC10104									
			15C15	16C66	17C93	206077	206059									
			15C25	16C85	17C95											
		L (*n* = 2)	16C58	206070				β, γ	−	−	−	−	−	−	−	−
		ND (*n* = 6)	16C29	17C61	17C78	17C72	17C79	γ	−	−	−	−	−	−	−	−
			17C53													
	II	B (*n* = 32)	PA20	16C57	17C45	16C56	17C87	α, γ	−	−	−	−	−	−	−	−
			PA28	16C60	17C46	17C106	17C89									
			15C20	16C61	17C50	17C80	19C16									
			15C23	16C90	17C54	XC2-1	19C20								−	−
			15V21	16C96	17C55	HG1-1	19C34									
			16C42	17C101	17C66	HG1-4	17C69									
			16C45	17C103											−	−
		J (*n* = 4)	15C10	15C12	16C82	17C70		γ	−	−	−	−	−	−	−	−
		K (*n* = 3)	17C51	17C84	19C19			γ	−	−	−	−	−	−	−	−
		M (*n* = 59)	PA7	PA48	17C56	SC2-1	206041	α, β, γ	−	−	−	−	−	−	−	−
			PA10	PA49	17C62	SC2-2	206045									
			PA17	15C02	17C63	SC2-2	206047								−	−
			PA19	15C04	17C65	SC2-3	206055									
			PA21	15C07	17C76	SC2-3	206061									
			PA26	15C13	19C02	SC2-4	206062								−	−
			PA29	15C14	19C09	XC2-2	206069									
			PA30	15C30	19C10	206107-2	206079									
			PA31	16C03	19C36	206109	206085								−	−
			PA35	16C102	206107-1	17C47	206105									
			PA36	PA37	PA39	PA46	PA47									
			16C20	16C22	16C64	16C69									−	−
		ND (*n* = 21)	PA18	17C57	17C81	17C94	206040	α, β, γ								
			PA22	17C60	17C82	17C97	206050									
			PA34	17C74	17C85	19C03	206058								−	−
			17C102	17C75	206102	17C99	17C98									
			17C104													
*Pseudomonas putida*			ST25-10					α	−	−	−	−	−	−	−	−
*Pseudomonas putida*			GIM1.57					α	−	−	−	−	−	−	−	−
*Pseudomonas fuscovaginae*			ST42-2					α	−	−	−	−	−	−	−	−
*Pseudomonas hunanensis*			0617-8					α	−	−	−	−	−	−	−	−
*Pseudomonas fulva*			0625-4					α	−	−	−	−	−	−	−	−
*Pseudomonas kilonensis*			ST38-5					α	−	−	−	−	−	−	−	−
*Pseudomonas lini*			M41023-1					α	−	−	−	−	−	−	−	−
*Pseudomonas jessenii*			ST42-4					α	−	−	−	−	−	−	−	−
*Pseudomonas pseudoalcaligenes*			GMCC1.1806					α	−	−	−	−	−	−	−	−
*Pseudomonas chlororaphis*			1143-3					α	−	−	−	−	−	−	−	−
*Pseudomonas fragi*			52532-7					α	−	−	−	−	−	−	−	−
*Pseudomonas mendocina*			CMCC1.1804					α	−	−	−	−	−	−	−	−
*Pseudomonas mosselii*			ST42-10					α	−	−	−	−	−	−	−	−
*Pseudomonas corrugata*			ST19-4					α	−	−	−	−	−	−	−	−
*Pseudomonas oleovorans*			M43075-4					α	−	−	−	−	−	−	−	−
*Pseudomonas taiwanensis*			0617-3					α	−	−	−	−	−	−	−	−
*Pseudomonas geniculata*			52023-3					α	−	−	−	−	−	−	−	−
*Pseudomonas fluorescens*			51184-3					α	−	−	−	−	−	−	−	−
*Pseudomonas fluorescens*			GIM1.492					α	−	−	−	−	−	−	−	−
*Escherichia coli*			25922					α	−	−	−	−	−	−	−	−
*Escherichia coli*			1656-1					α	−	−	−	−	−	−	−	−
*Staphylococcus hominis*			1006-1					α	−	−	−	−	−	−	−	−
*Staphylococcus hominis*			0656-4					α	−	−	−	−	−	−	−	−
*Staphylococcus haemolyticus*			0629-2					α	−	−	−	−	−	−	−	−
*Staphylococcus aureus*			ATCC 22923					α	−	−	−	−	−	−	−	−
*Staphylococcus aureus*			522					α	−	−	−	−	−	−	−	−
*Salmonella*			837					α	−	−	−	−	−	−	−	−
*Salmonella*			926					α	−	−	−	−	−	−	−	−
*Yersinia enterocolitica*			y2602					α	−	−	−	−	−	−	−	−
*Yersinia enterocolitica*			y3585					α	−	−	−	−	−	−	−	−
*Listeria monocytogenes*			1333-2					α	−	−	−	−	−	−	−	−
*Listeria monocytogenes*			2545-2					α	−	−	−	−	−	−	−	−
Total		254														

*a: CMCC, china Medical culture collection, China. b: ATCC, american type culture collection, United States. c: GIM, guangdong institute of microbiology, China. d: α, the guangdong institute of microbiology, China; β, guangdong huankai Co., Ltd., China; γ, zhujiang hospital, Guangzhou, China. Result (±) indicate positive and negative signals.

### Evaluation of the Specificity and Sensitivity of Candidate Target Genes

Primer Premier software (version 6.0) was used to design primers for species-specific targets of *P. aeruginosa* serogroup G. Primer sequences are listed in Table 2. The specificity of each primer was assessed against the bacterial sequences listed in Table 1. Each 20 μL PCR mixture consisted of 10 μL of 2 × Taq Master mix (Novoprotein Scientific, Shanghai, China), 0.5 μL of forward and reverse primers (10 μM), 2 μL of target DNA template, and sterile distilled water (to a final volume of 20 μL). The PCR conditions used were 98°C for 3 min, followed by 35 cycles of denaturation at 95°C for 30 s, annealing at 60°C for 30 s, elongation at 72°C for 1 min, and final elongation at 72°C for 10 min. The products were analyzed using 2% agarose gel electrophoresis and visualized using GoldView^®^ staining (0.01%, v/v) under ultraviolet light.

**TABLE 2:** Specific target genes and primers are used for the detection of *P. aeruginosa* serogroup G.

Gene	*Name of target genes	Primer set name	Sequences (5′–3′)	Product size (bp)	Serotype specificity
*group_40682*	*PA59_01276*	G-PCR-1	For: CTGTTTTCGCTATTATTTATCTTCG	278	G (+)
			Rev: AAAACACACAAACAATCAAAAATC		
		G-real-time PCR	For: ACTTCCCATCCCTGTAACCCT	133	G (+)
			Rev: CGGCCAGACTGCTTCCATA		
*wzzB*	*PA59_01887*	G-PCR-4	For: TCCCTGAGATGGCTGATTG	450	G (+)
			Rev: CTGCATGGAACGCTTGACT		
*wbpA*	*PA59_01888*	G-PCR-5	For: GTTGGTCTTCCGCTTGCTG	342	G (+)
			Rev: GTCTTCTTCCGTCGCTCCC		
*group_234447*	*PA59_01891*	G-PCR-7	For: CGTTATCTGCCTGAGTGTA	459	G (+)
			Rev: GAAAGTAAGCGTCAGTGGTG		
*epsF_4*	*PA59_01894*	G-PCR-9	For: GATTGCCTTGTATTACCACTG	116	G (+)
			Rev: AATGAGATCCTCGATACCTTT		
*group_234710*	*PA59_04268*	G-PCR-10	For: CTGTTTTCGCTATTATTTATCTTCG	298	G (+)
			Rev: GAAAACACACAAACAATCAAAAATC		
*group_71614*	*PA59_01892*	G-PCR-13	For: TTCGGTCAGTTCGGTAGGC	324	G (+)
			Rev: ATCATCGGCAAGAGGCATT		
*gnu*	*PA59_01896*	G-PCR-14	For: CCAAACGGGAAGCGGAGCA	410	G (+)
			Rev: GCACAGGCGGCGAGCAAAT		

*Reference strain is P. aeruginosa PA59. The reference gene is GCA_009497675.1_ASM949767v1. Result (+/−) indicate positive and negative signals.

Ten-fold serial dilutions of *P. aeruginosa* serogroup G strain PA206052 (10^8^ to 10^1^ CFU/mL) were subjected to DNA extraction as described above. For each dilution, 2 μL was used as a template for PCR amplification. The target gene with the highest detection limit was selected for further experiments.

### Real-Time Polymerase Chain Reaction Conditions for the Detection of *P. aeruginosa* Serogroup G

The total reaction volume was 20 μL, including 10 μL of TB Green™ Premix Ex Taq™ II (TaKaRa, Biotech, Dalian, China), 1 μL each of forward and reverse primers (10 μM), 6 μL of sterile water, and 2 μL of purified bacterial genomic DNA as the template. A Light Cycler 96 System (Roche, Switzerland) was used for thermal cycling as follows: denaturation at 95°C for 60 s, followed by 40 cycles of denaturation at 95°C for 10 s, and annealing at 60°C for 30 s. Real-time PCR was performed in triplicate, with parallel analysis in 96-well plates. The DNA template was substituted with sterile water in the negative controls to ensure the absence of contaminants.

### Evaluation of Real-Time Polymerase Chain Reaction Specificity and Co-infection-Related Interference

Genomic DNA from 13 *P. aeruginosa* strains and 15 other bacterial strains was used as a template for real-time PCR analysis to evaluate the specificity of the assay (Supplementary Table 4). To further assess the potential interference caused by co-infection, *P. aeruginosa* serogroups D and F (interfering bacteria) were mixed with the sample. The target serogroup G was cultured for 12 h, and the initial concentration of each bacterial suspension was determined using the plate count method. The concentration of the target bacterium, serogroup G, was adjusted to 10^4^ CFU/mL, and the interfering bacteria were diluted to 10^8^–10^1^ CFU/ml. The cultures of *P. aeruginosa* serogroup G cultures were mixed with those of the other serotypes at ratios (D or F to G) of 10^4^:1, 10^3^:1, 10^2^:1, 10:1, 1:1, 1:10, 1:10^2^, and 1:10^3^. Purified DNA was extracted from the mixtures, as described in section “Mining of *P. aeruginosa* Serogroup G-Specific Targets,” and used as a template for real-time PCR.

### Detection of *P. aeruginosa* Serogroup G in Artificially Inoculated Bottled Drinking Water

Bottled drinking water samples, purchased from a local supermarket, autoclaved at 121°C/0.1 MPa for 15 min, were negative for *P. aeruginosa*, as assessed using the traditional culture method (Marei, 2020). Briefly, 1 mL of each bottled drinking water sample was added to 9 mL of saline solution to obtain the matrix. Different concentrations of the PA206052 culture were inoculated into the matrix, yielding final bacterial concentrations ranging from 4.0 × 10^8^ CFU/mL to 4.0 × 10^1^ CFU/ml. Subsequently, 1 ml of the suspension containing the strain PA206052 strain collected from each sample was subjected to DNA extraction. Genomic DNA (50 ng) was subsequently used for PCR and real-time PCR analysis. The amplification systems and procedures are described in Sections “Evaluation of the Specificity and Sensitivity of Candidate Target Genes” and “Real-time Polymerase Chain Reaction Conditions for the Detection of *P. aeruginosa* Serogroup G.”

### Testing of Natural Water Samples

Thirty-seven aquatic samples (from the surrounding living environment and random from tributaries of the Pearl River Basin) were collected to validate the efficacy of the real-time PCR method. The water samples were tested for the presence of *P. aeruginosa* serogroup G using the traditional culture method and the slide agglutination method using *P. aeruginosa* antisera (Denka Seiken, Tokyo, Japan). *P. aeruginosa* contamination in water is usually low; therefore, an enrichment procedure was employed before PCR analysis. Briefly, a water sample (250 mL) was filtered through a 0.45 μm membrane (Millipore Co., Billerica, MA, United States) in a stainless-steel multi-line filter system (Huankai Co., Guangzhou, China). The membrane was placed in LB broth at 37°C for 12 h. Whole-cell DNA was extracted using a bacterial genomic DNA purification kit (Omega Bio-Tek Inc., Norcross, GA, United States), according to the manufacturer’s instructions. For each sample, 1 mL of the LB enrichment culture was collected. Genomic DNA was extracted from the LB enrichment cultures for PCR and real-time PCR assessment.

## Results

### Mining for *P. aeruginosa* Serogroup G-Specific Target Sequences

A total of 343 *P. aeruginosa* strain genome assemblies were downloaded from the NCBI Genome bank (last accessed on January 31, 2022). The complete genome (GCA_009497675.1_ASM949767v1) of *P. aeruginosa* serogroup G, PA59 strain (CP024630.1), retrieved from the GenBank database was used as a reference sequence. Candidate serogroup G-specific target sequences were obtained *via* pan-genome analysis the *P. aeruginosa* strain sequences. All candidate target sequences were further searched against the NCBI databases using the online BLAST program based on sequence similarity with phylogenetically connected or aloof species (Supplementary Figure 1). Fourteen candidate serogroup G-specific targets existed only in serogroup G strains (Supplementary Table 2); the nucleic acid sequences of the targets are shown in Supplementary Table 2. One of gene, *PA_01889*, has been reported in the literature (Li et al., 2018). When whole genome sequence was used to mine G-specific target genes, the number of interspecific gene sequences with low homology of *P. aeruginosa* may have little effect on the number of serum-specific targets obtained (Supplementary Table 5).

### Screening Specific Gene Targets for *P. aeruginosa* Serogroup G

The specificity of the target sequences that were obtained through comparative genomic analysis was tested *via* PCR. Primers with specific target genes determined by experiments are shown in Table 2. The specific primers are G-PCR-1, G-PCR-4, G-PCR-5, G-PCR-7, G-PCR-9, G-PCR-10, G-PCR-13, and G-PCR-14. The novel specificity genes targets, *PA59_01276*, *PA59_01887*, *PA59_01888*, *PA59_01891*, *PA59_01894*, *PA59_04268*, *PA59_01892*, and *PA59_01896*, for all 18 *P. aeruginosa* serogroup G strains were detected at 100 and 100% exclusivity for the 204 other serogroups strains of *P. aeruginosa* and 24 other species (Table 1).

Four of these genes encode known proteins: one gene *PA59_01887* (*wzzB*) codes Chain length determinant protein, one gene *PA59_01888* (*wbpA*) regulates UDP-N-acetyl-D-glucosamine 6-dehydrogenase, one gene *PA59_01884 (epsF_4)* related to the Putative glycosyltransferase EpsF operation, and the gene *PA59_01889 (gun*) N-acetyl-alpha-D-glucosaminyl-diphospho-ditrans, octacis-undecaprenol 4-epimerase. The remaining four genes: *PA59_01891 (group_234447)*, *PA59_01891 (group_40682)*, *PA59_01891 (group_71614)*, *PA59_04268 (group_234710)*, encode unknown protein (Supplementary Table 2).

### Target Gene Sensitivity Evaluation

**FIGURE 2:**
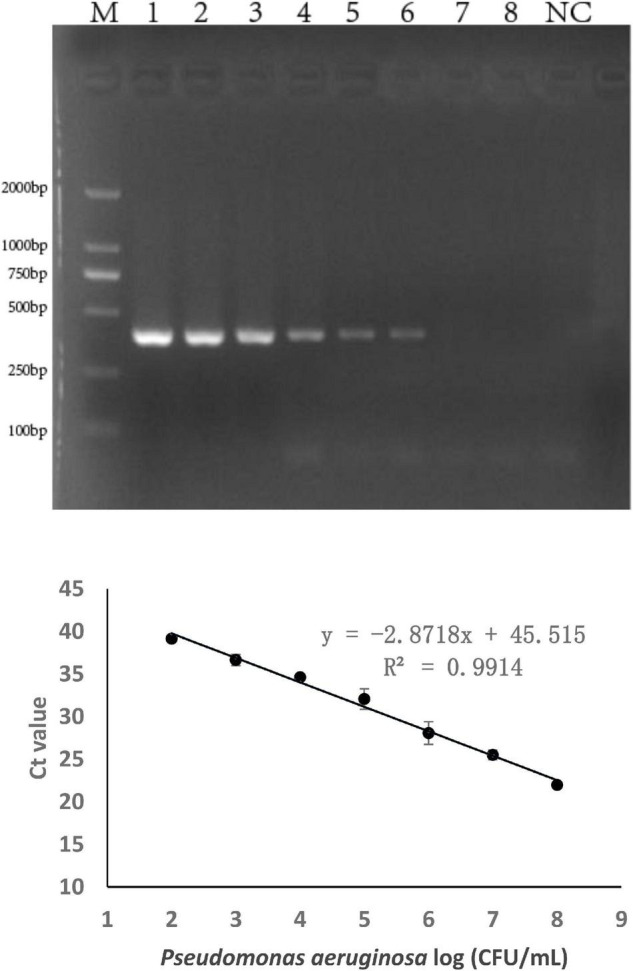
PCR detection sensitivity using primers for *P. aeruginosa* serogroup G-specific genes. 2.1: Amplification using *PA59_01276* primers. Lane M: DL DNA 2000 marker, lane NC: negative control, lanes 1–8: Ten-fold sample dilutions (4.0 × 10^8^ CFU/mL to 4.0 × 10^1^ CFU/mL). 2.2: Establishment of a standard curve for the detection of *P. aeruginosa* serogroup G. Plots of Ct values against the log numbers of *P. aeruginosa* (4.0 × 10^2^ CFU/mL–4.0 × 10^8^ CFU/mL) in pure culture.

The sensitivity of the specific genes was further verified by PCR amplification using *P. aeruginosa* serogroup G strain PA 206052. The lower limit of PCR detection ranged between 10^3^ and 10^5^ CFU/mL for pure culture (Supplementary Table 3). However, when the same concentration of serogroup G genomic DNA was used as a template for PCR amplification, the bands that were amplified using with the *PA59_01276* primers were brighter than those that were amplified using with the other primers. Based on these results, combine with the LOD and product size results, we chose targeting gene *PA59_01276* set for our real-time PCR assay for *P. aeruginosa* serogroup G detection. Thus, the *PA59_01276* primer set was chosen for further experiments. We established a real-time PCR approach; the linear regression equation was y = −2.8718x + 45.515 (*R*^2^ = 0.9914), and the detection limit for pure *P. aeruginosa* serogroup G was 10^2^ CFU/mL (Figure 2).

### Specificity and Anti-interference Detection Using the Real-Time Polymerase Chain Reaction Assay

**FIGURE 3:**
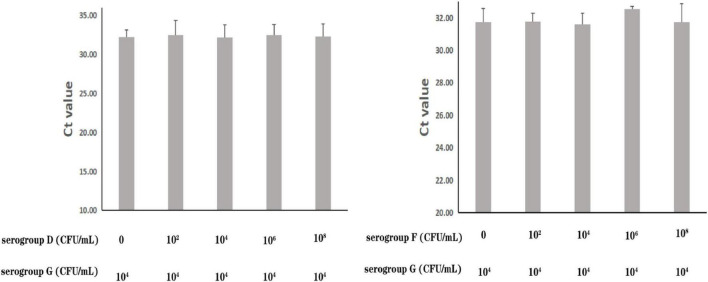
Assessment of interference with the real-time PCR-based detection of *P. aeruginosa* serogroup G by co-infection with other serogroups. Detection of *P. aeruginosa* serogroup G (4.0 × 10^4^ CFU/mL) in the presence of *P. aeruginosa* serogroup D (0, 4.7 × 10^2^ CFU/mL, 4.7 × 10^4^ CFU/mL, 4.7 × 10^6^ CFU/mL, 4.7 × 10^8^ CFU/mL, respectively) (3.1) and serogroup F (0, 2.4 × 10^2^ CFU/mL, 2.4 × 10^4^ CFU/mL, 2.4 × 10^6^ CFU/mL, 2.4 × 10^8^ CFU/mL, respectively) (3.2).

The specificity of the real-time PCR method based on *PA59_01276* primers was checked in 13 *P. aeruginosa* and 16 other bacterial species (Supplementary Table 4). DNA amplification showed exclusivity for *P. aeruginosa* serogroup G. To further assess the precision of its susceptibility and interference, *P. aeruginosa* serogroup G strain PA206052 was mixed with other serotypes at various proportions. All amplifications showed near cycle threshold (Ct) values (Figure 3), irrespective of the target to interfering strain proportion, indicating that the presence of serotypes F and D did not interfere with serogroup G detection.

### Detection of *P. aeruginosa* Serogroup G in Artificially Contaminated Bottled Drinking Water *via* Polymerase Chain Reaction and Real-Time Polymerase Chain Reaction

**FIGURE 4:**
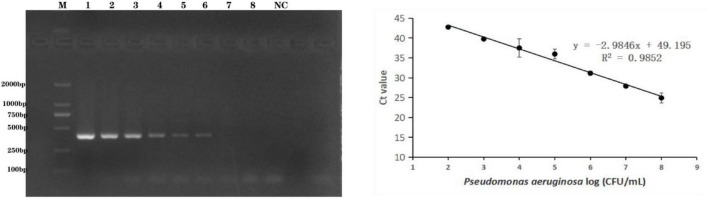
Practical application for the detection of *P. aeruginosa* serogroup G in spiked drinking water samples. 4.1: Amplification of PCR assay using *PA59_01276* primers. Lanes 1–8: Samples spiked with 10-fold dilutions of serogroup G (4.0 × 10^8^ CFU/mL–4.0 × 10^1^ CFU/mL), lane M: DL DNA 2000 marker; lane NC: negative control. 4.2: Amplification of real-time PCR assay using *PA59_01276* primers. Plots of Ct values against the log numbers of *P. aeruginosa* (4.0 × 10^2^ CFU/mL–4.0 × 10^8^ CFU/mL) in artificially contaminated drinking water.

The methods were applied to the detection of *P. aeruginosa* serogroup G in artificially contaminated bottled drinking water samples. *P. aeruginosa* (4.0 × 10^8^ to 4.0 × 10^1^ CFU/mL) was added to the sample, and real-time PCR and end-point PCR were used to detect serogroup G in the spiked samples. As shown in Figure 4, the detection limit in the artificially bottled drinking water samples was 4.0 × 10^4^ CFU/mL, detected using end-point PCR. The real-time PCR detection conditions were further optimized to establish a standard curve of detection quantity of the *P. aeruginosa* G serum group. The linear detection range of this method was 4.0 × 10^8^ CFU/mL to 4.0 × 10^3^ CFU/mL (Figure 4), and the linear regression equation was y = −2.9846x + 49.195 (*R*^2^ = 0.9852). The limit of detection (LOD) of the novel target-based real-time PCR assay was calculated as 4.0 × 10^3^ CFU/mL for the *P. aeruginosa* serogroup G. In comparison with the end-point PCR approach, and the real-time PCR approach was more sensitive by order of magnitude.

### Detection of *P. aeruginosa* Serogroup G in Water Samples

As determined *via* the traditional culture approach and slide agglutination using *P. aeruginosa* antisera, three of the 37 samples (sample numbers 25, 30, and 37) collected from the surrounding living surroundings and markets were contaminated with *P. aeruginosa* serogroup G. These results were consistent with those obtained using real-time PCR and PCR. After three parallel tests were carried out using the samples, the mean average Ct value for *P. aeruginosa* serogroup G detection was 20.87, 28.26, and 25.68, respectively (Table 3 and Supplementary Figure 2).

**TABLE 3:** Culture-based identification of *P. aeruginosa* serogroup G, real-time PCR and PCR assay results from 37 water samples.

No.	Sample names	Culture identification and slide agglutination	Real-time PCR result			Culture-based slide agglutination	PCR results
			Parallel test 1	Parallel test 2	Parallel test 3		
1	Mineral water	Negative	−	−	−	−	−
2	Mineral water	Negative	−	−	−	−	−
3	Mineral water	Negative	−	−	−	−	−
4	Bottled water	Negative	−	−	−	−	−
5	Bottled water	Negative	−	−	−	−	−
6	Bottled water	Negative	−	−	−	−	−
7	Surface water	Negative	−	−	−	−	−
8	Surface water	Negative	−	−	−	−	−
9	Surface water	Negative	−	−	−	−	−
10	Surface water	Negative	−	−	−	−	−
11	Surface water	Negative	−	−	−	−	−
12	Surface water	Negative	−	−	−	−	−
13	Surface water	Negative	−	−	−	−	−
14	Surface water	Negative	−	−	−	−	−
15	Surface water	Negative	−	−	−	−	−
16	Surface water	Negative	−	−	−	−	−
17	Surface water	Negative	−	−	−	−	−
18	Surface water	Negative	−	−	−	−	−
19	Surface water	Negative	−	−	−	−	−
20	Surface water	Negative	−	−	−	−	−
21	Drinking water	Negative	−	−	−	−	−
22	Drinking water	Negative	−	−	−	−	−
23	Drinking water	Negative	−	−	−	−	−
24	Drinking water	Negative	−	−	−	−	−
25	Drinking water	*P. aeruginosa* serogroup G	20.97	20.93	20.71	+	+
26	Drinking water	Negative	−	−	−	−	−
27	Drinking water	Negative	−	−	−	−	−
28	Drinking water	Negative	−	−	−	−	−
29	Drinking water	Negative	−	−	−	−	−
30	Drinking water	*P. aeruginosa* serogroup G	28.08	28.41	28.28	+	+
31	Drinking water	Negative	−	−	−	−	−
32	Drinking water	Negative	−	−	−	−	−
33	Drinking water	Negative	−	−	−	−	−
34	Drinking water	Negative	−	−	−	−	−
35	Drinking water	Negative	−	−	−	−	−
36	Air conditioning condensate	Negative	−	−	−	−	−
37	Air conditioning condensate	*P. aeruginosa* serogroup G	25.92	25.36	25.75	+	+

## Discussion

Serological typing is one of the most ordinarily applied phenotypic identification approaches for the classification of *P. aeruginosa* isolates. In epidemiological research, it is usually needed to quickly trace the transmission of *P. aeruginosa* by combining molecular typing and classical serotype characteristics. Molecular typing methods commonly include random amplified polymorphic DNA analysis, pulsed-field gel electrophoresis, and multilocus sequence analysis (Toennies et al., 2021). Other approaches for detecting and identifying *P. aeruginosa*, such as PCR-based open reading frame typing, whole-genome sequencing, and single nucleotide polymorphism typing, are based on genes specific for *P. aeruginosa* serotypes (Hao et al., 2015; Thrane et al., 2016; Huszczynski et al., 2020). However, thus far, previous studies on detection targets used to identify *P. aeruginosa* serogroup were performed on a small scale, and the existing approaches for distinguishing serum types are not suitable for separating closely related serum types since their sequences are similar between different serum strains. Consequently, it is required to provide specific molecular targets for serotyping new *P. aeruginosa* strains and quickly identify different serotypes.

The rapid development of computing and genomics has improved efficiency and personalized serotype-specific target genome mining in recent years. Comparative genomics was chosen because it includes the powerful capabilities of the software, and has the advantage of whole-genome sequences availability, thereby providing more information than a single gene or coding sequence to identify serotype-specific targets. Based on the whole-genome sequencing technology, researchers established a clinical serotyping method, analyzed the assembled input genome through BLASTN, compared the sequence with the OSA cluster database, queried the genome coverage, and classified the OSA cluster with a coverage of more than 95% as serogroup-positive (Thrane et al., 2016). In another serological typing method, the investigator selected a reference genome, divided it into 1000 bp segments in a silica gel, and compared them to all other genome sequences to obtain details of serogroup specificity (Shang et al., 2021). To increase the possibility of identifying specific sequences or gene spacer regions across two genes, researchers used a new comparative genomics method and screened nucleotide sequences for *Salmonella* serogroup-specific detection (Liu et al., 2011; Yu et al., 2011). However, based on little strain information or the limited number of genomes in the public domain, even if many candidate target sequences are obtained, the verification process is very time-consuming and not suitable for practical applications (Yu et al., 2011). The whole-genome comparisons have been used to discover specific markers in bacteria, such as antibiotic resistance, quorum sensing, biofilm-forming, virulence, and serotype, all of which are ordinarily connected to genes that are acquired from other species through horizontal gene transfer (Thrane et al., 2016; Medina-Rojas et al., 2020; Karash et al., 2021; Mahto et al., 2021; Spinler et al., 2022). In this study, a whole-genome approach was used to identify markers with high reliability and specificity.

To obtain highly feasible and reliable targets, we established a database of 343 strains of *P. aeruginosa*, which revealed eight serogroup G-specific novel targets. Interestingly, the specific genes were designated related to enzyme and protein coding sequences. In addition to being potential serotype-specific targets, these serotype-specific hypothetical protein-coding regions may also help to analyze the relationship between gene structure and function in the future, to improve the understanding of the unique metabolic behavior of *P. aeruginosa* serogroup G. Furthermore, recent strategies to expand antibiotic diversity have aimed for exploiting new targets that were identified through genomic approaches (Mills, 2006; Payne et al., 2007). Essential gene codes for antibiotic targets are usually identified *via* whole genome sequencing, and serum targets established using this method may provide targets for the discovery of new antibiotics. More likely, it will provide a basis for the development of a vaccine based on serogroup G of *P. aeruginosa*.

Excellent specificity and sensitivity of molecular methods are important for the rapid identification of microorganisms. *P. aeruginosa* has two antigens, “O” = somatic and “H” = flagellar. Serotyping in most epidemiological studies is done using “O” antigens. Usually, *P. aeruginosa* produces two distinct forms of O-antigens, namely, a common polysaccharide antigen (CPA, A-band) composed of D-rhamnose homopolymers and an OSA consisting of a heteropolymer with three to five distinct sugars in its repeat units (Lam et al., 2011; Nasrin et al., 2022). The OSA determines the serotype specificity of the bacterium and thus differentiates the *P. aeruginosa* serotype (Nasrin et al., 2022). Researchers believed that a certain correlation exists between O antigen serotype and toxin secretion. *P. aeruginosa* may secrete four toxins, including *ExoS* (exoenzyme S), *ExoT* (exoenzyme T), *ExoU* (cytotoxin), and *ExoY* (Catho et al., 2021). However, some clinical isolates belonging to the serogroup G do not secrete any of the four toxins (Koch et al., 2014; Kuo et al., 2020; Catho et al., 2021). Therefore, the toxin-dependent method is not reliable for serological typing. PCR methods have been used to distinguish the serogroup and serotype of *P. aeruginosa* (Parsons et al., 2002; Allison and Castric, 2016). The most frequent serogroup G (17.8%) of *P. aeruginosa* was identified using the OSA cluster database screening method (Del Barrio-Tofino et al., 2019). Based on the genes *wbpP* and *ihfB*, PCR methods have been used to distinguish the serogroup G of *P. aeruginosa* (Koch et al., 2014; Li et al., 2018; Richard et al., 2020). Interestingly, we found that the gene *wbgU_1* (*PA59_01889*) we mined has the same base sequence as target *wbpP* (*PA59_01889*) reported in the literature, they are the same target gene. However, the gene *ORF_14* was not serogroup G-specific. The coverage of *ORF_14* gene was 96.7% in the target serum of serogroup G, non-specific amplification was observed in non-target serum 1.4% (4/281) (Table 4). Essentially, the specificity of molecular targets is very important for serum typing, especially to detect *P. aeruginosa* serogroup in the context of extremely complex food substrates. After verification, the new molecular detection targets excavated in this study covered 100% of the target serum group G strains but did not exist in the non-target strains. Therefore, the detection targets obtained in this study for *P. aeruginosa* serogroup G by pan-genome analysis display a better specificity to meet the needs of food safety and water testing.

**TABLE 4:** Presence profile of novel *P. aeruginosa* serogroup G-specific targets for target and non-target strains.

Genes	Serogroup	Primer information	Related gene	Presence profile		Source
				In target	In non-target	
*group_40682*	monovalent serogroup G	G-PCR-1	*PA59_01276*	62 (100%)	0	This study
*wzzB*	monovalent serogroup G	G-PCR-4	*PA59_01887*	62 (100%)	0	This study
*wbpA*	monovalent serogroup G	G-PCR-5	*PA59_01888*	62 (100%)	0	This study
*wbgU_1*	monovalent serogroup G	G-PCR-6	*PA59_01889*	62 (100%)	0	This study
*group_234447*	monovalent serogroup G	G-PCR-7	*PA59_01891*	62 (100%)	0	This study
*epsF_4*	monovalent serogroup G	G-PCR-9	*PA59_01894*	62 (100%)	0	This study
*group_234710*	monovalent serogroup G	G-PCR-10	*PA59_04268*	62 (100%)	0	This study
*group_71614*	monovalent serogroup G	G-PCR-13	*PA59_01892*	62 (100%)	0	This study
*gnu*	monovalent serogroup G	G-PCR-14	*PA59_01896*	62 (100%)	0	This study
*ORF_14*	monovalent serogroup G (O6)	*AF498417*	*PA59_01897*	60 (96.7%)	4 (1.4%)	Koch et al., 2014
*wbpP*	monovalent serogroup G (O6)	*AC104736*	*PA59_01889*	62 (100%)	0 (0%)	Li et al., 2018

The real-time PCR method used in this study targets novel serogroup-specific genes, which helps identify *P. aeruginosa* serogroup G distinctly from interfering serogroups, such as serogroup D and F, even when the interfering serogroup is more abundant than serogroup G in the sample. The limit of real-time PCR detection for *P. aeruginosa* serogroup G in a pure culture medium using primers for *PA59_01276* was 1.12 × 10^2^ CFU/mL and 4.0 × 10^3^ CFU/mL in food samples contaminated with *P. aeruginosa*. In a previous study, PCR amplification of *wbpP* had a limit of ≥10^3^ CFU/mL, and *ihfB* had a limit of ≥10^5^ CFU/mL for *P. aeruginosa* serogroup G (Wu et al., 2016). Therefore, our real-time PCR approach is 1–3 orders of magnitude more sensitive than the previously reported method, indicating that we successfully detected *P. aeruginosa* serogroup G. These values are comparable to those obtained using most PCR methods applied to detect microorganisms in water without prior enrichment (Goepfert et al., 2020). This method has a good consistency for the detection of *P. aeruginosa* serogroup G without interference from non-target bacteria and actual samples. The results indicated that the chosen target had a strong anti-interference capability and excellent specificity and could quickly, sensitively, and accurately detect *P. aeruginosa* serogroup G in artificially contaminated samples. The newly mined targets were used to explore the spread of *P. aeruginosa* serogroup G successfully in actual contaminated water samples.

In addition, the total analysis time, including enrichment culture, sample preparation, genomic DNA extraction, and real-time PCR amplification, took less than 15 h. Compared to the traditional serological typing approach, which requires at least 5 days to complete, the time to obtain results using our method is significantly shorter than that of the conventional serological typing method. At the same time, the cost of real-time PCR is low. Ideally, the cost is only a tenth of traditional typing methods (from $50 for traditional typing methods to $5 for real-time PCR method).

## Conclusion

In conclusion, following the analysis of the whole-genome sequences analysis of different *P. aeruginosa* serogroups from the GenBank, we obtained eight specific detection targets, *PA59_01276*, *PA59_01887*, *PA59_01888*, *PA59_01891*, *PA59_01894*, *PA59_04268*, *PA59_01892*, and *PA59_01896*, of monovalent serogroup G by comparative genomics. Based on the novel molecular target gene *PA59_01276*, we established a real-time fluorescence quantitative PCR approach for the speedy detection and identification of G serotypes. The real-time PCR approach is specific, sensitive, and has a powerful anti-interference ability, with a detection limit of 4.0 × 10^2^ CFU/mL in pure culture and 4.0 × 10^3^ CFU/mL in artificially contaminated drinking water samples. In addition, the 37 actual sample detection for PCR and real-time PCR exhibits satisfactory results. It provides epidemiological investigations with a powerful tool to assess *P. aeruginosa* contamination for water testing and improving food safety. Moreover, the targets novel serogroup-specific genes that were obtained through this approach can provide a basis for the development of new antibiotics and may promote the development of the *P. aeruginosa* G serum vaccine.

## Data Availability Statement

The original contributions presented in this study are included in the article/Supplementary Material, further inquiries can be directed to the corresponding author/s.

## Author Contributions

CW contributed to investigation, methodology, data curation, and writing original draft. QY contributed to the project administration and data curation. YD, QG, RP, and HZ contributed to the data curation. JZ contributed to the supervision and resources. JW and QW contributed to the supervision and writing review and editing. All authors contributed to the article and approved the submitted version.

## Conflict of Interest

The authors declare that the research was conducted in the absence of any commercial or financial relationships that could be construed as a potential conflict of interest.

## Publisher’s Note

All claims expressed in this article are solely those of the authors and do not necessarily represent those of their affiliated organizations, or those of the publisher, the editors and the reviewers. Any product that may be evaluated in this article, or claim that may be made by its manufacturer, is not guaranteed or endorsed by the publisher.

## Footnotes

1
https://www.ncbi.nlm.nih.gov/genome/

2
https://blast.ncbi.nlm.nih.gov/Blast.cgi

## Abbreviations

CFU: colony-forming units
Ct: cycle threshold
LB: Luria–Bertani
NCBI: National Center for Biotechnology Information
PCR: polymerase chain reaction
OSA: O-specific antigen
LOD: limit of detection.
