# Supplementary material for: Detection of Pseudomonas aeruginosa Serogroup G Using Real-Time PCR for Novel Target Genes Identified Through Comparative Genomics
Source: Front Microbiol. 2022 Jun 24;13:928154. doi: 10.3389/fmicb.2022.928154 (PMC9263582; doi:10.3389/fmicb.2022.928154)
Supplement: Supplementary file 1 [file Data_Sheet_1.docx]

**Supplementary Material**

**Detection of *Pseudomonas aeruginosa*** **serogroup G using real-time PCR for novel target genes identified through comparative genomics**

Chufang Wang ^a, b, c #^, Qinghua Ye ^b, c^, Yu Ding ^b, c^, Jumei Zhang ^b, c^, Qihui Gu ^b, c^, Rui Pang ^b, c^, Hui Zhao ^b, c^, Juan Wang ^a, b, c*^, Qingping Wu ^a, b, c*^

^a^ College of Food Science, South China Agricultural University, Guangzhou, 510642, China

^b^ Guangdong Provincial Key Laboratory of Microbial Safety and Health, State Key Laboratory of Applied Microbiology Southern China, Institute of Microbiology, Guangdong Academy of Sciences, Guangzhou, China

^c^ Key Laboratory of Agricultural Microbiomics and Precision Application, Ministry of Agriculture and Rural Affairs.

**^*^ Corresponding author:**

Professor Qingping Wu

1. mail: [wuqp203@163.com](mailto:wuqp203@163.com)

Tel: +86-20-87688132; fax: +86-20-87688132

Address: Institute of Microbiology, Guangdong Academy of Sciences, Yard 100#, Xianlie Zhong Road, Yuexiu District, 510070 Guangzhou, P.R.China.

Associate Professor Juan Wang

E-mail: wangjuan@scau.edu.cn

Address: College of Food Science, South China Agricultural University, Guangzhou 510642, China.

^#^ This author contributed to the manuscript.

**Table S1.** Information for monovalen serogroup of *P. aeruginosa* isolates analyzed during this study.

| Bacterial species | Polyvalent  serogroup | Monovalen  serogroup | Accession no. | | | |
| --- | --- | --- | --- | --- | --- | --- |
| *P. aeruginosa* | I (20 ) | A | GCA_000568235.2 | GCA_001420205.1 | GCA_016762735.1 |  |
|  |  | C | GCA_001444745.1 | GCA_001444765.1 | GCA_019430035.1 | GCA_019430235.1 |
|  |  | H | GCA_001444835.1 | GCA_013375815.1 |  |  |
|  |  | I | GCA_001420225.1 | GCA_019430415.1 | GCA_021441565.1 | GCA_021533055.1 |
|  |  | L | GCA_001444975.1 | GCA_003032395.1 | GCA_019430585.1 | GCA_019430625.1 |
|  |  |  | GCA_001756495.1 | GCA_001756515.1 | GCA_001756585.1 |  |
|  | II (11 ) | B | GCA_001420185.1 | GCA_001420265.1 | GCA_001444895.1 | GCA_019429735.1 |
|  |  |  | GCA_019430085.1 | GCA_019430435.1 | GCA_019430465.1 | GCA_019430635.1 |
|  |  | J | GCA_001444865.1 |  |  |  |
|  |  | K | GCA_001444845.1 | GCA_001444995.1 |  |  |
|  | III (107) | D | GCA_001444755.1 | GCA_001444815.1 | GCA_013375755.1 | GCA_019430355.1 |
|  |  | E | GCA_001444955.1 | GCA_019429725.1 | GCA_019429935.1 | GCA_019430165.1 |
|  |  |  | GCA_002085755.1 | GCA_019429765.1 | GCA_019429985.1 | GCA_019430175.1 |
|  |  |  | GCA_011634585.1 | GCA_019429825.1 | GCA_019430105.1 | GCA_019430305.1 |
|  |  |  | GCA_013375875.1 | GCA_019429885.1 | GCA_019430125.1 | GCA_019430315.1 |
|  |  |  | GCA_019430385.1 | GCA_019430535.1 | GCA_019430665.1 |  |
|  |  | F | GCA_001420245.1 | GCA_019429845.1 | GCA_019430025.1 | GCA_019430405.1 |
|  |  |  | GCA_013375795.1 | GCA_019429865.1 | GCA_019430145.1 | GCA_019430505.1 |
|  |  |  | GCA_019324825.1 | GCA_019429875.1 | GCA_019430225.1 | GCA_019430515.1 |
|  |  |  | GCA_019429705.1 | GCA_019429925.1 | GCA_019430265.1 | GCA_019430565.1 |
|  |  |  | GCA_019429785.1 | GCA_019430005.1 | GCA_019430275.1 | GCA_019430605.1 |
|  |  |  | GCA_019430685.1 |  |  |  |
|  |  | G | GCA_021676015.1 | GCA_013375775.1 | GCA_013375915.1 | GCA_019430345.1 |
|  |  |  | GCA_004685005.1 | GCA_013375835.1 | GCA_016461995.1 | GCA_019430485.1 |
|  |  |  | GCA_009497675.1 | GCA_013375855.1 | GCA_019430065.1 | GCA_902703215.1 |
|  |  |  | GCA_013375755.1 | GCA_013375895.1 | GCA_019430205.1 | GCA_021730475.1 |
|  |  |  | GCA_013375775.1 | GCA_021675655.1 | GCA_021676015.1 | GCA_021676315.1 |
|  |  |  | GCA_013375835.1 | GCA_021675675.1 | GCA_021676055.1 | GCA_021676335.1 |
|  |  |  | GCA_013375855.1 | GCA_021675695.1 | GCA_021676075.1 | GCA_021676355.1 |
|  |  |  | GCA_013375895.1 | GCA_021675715.1 | GCA_021676115.1 | GCA_021676375.1 |
|  |  |  | GCA_013375915.1 | GCA_021675735.1 | GCA_021676155.1 | GCA_021676395.1 |
|  |  |  | GCA_021675535.1 | GCA_021675755.1 | GCA_021676175.1 | GCA_021676415.1 |
|  |  |  | GCA_021675555.1 | GCA_021675795.1 | GCA_021676195.1 | GCA_021676435.1 |
|  |  |  | GCA_021675575.1 | GCA_021675815.1 | GCA_021676215.1 | GCA_021676475.1 |
|  |  |  | GCA_021675595.1 | GCA_021675875.1 | GCA_021676235.1 | GCA_021676495.1 |
|  |  |  | GCA_021675615.1 | GCA_021675955.1 | GCA_021676255.1 | GCA_021676515.1 |
|  |  |  | GCA_021675635.1 | GCA_021675975.1 | GCA_021676275.1 | GCA_021676535.1 |
|  |  |  | GCA_021675995.1 | GCA_021676295.1 |  |  |
|  |  | O17 | GCA_001444915.1 |  |  |  |
|  |  | O18 | GCA_001444925.1 |  |  |  |
|  |  | O19 | GCA_001445005.1 |  |  |  |
|  |  | O20 | GCA_001445035.1 |  |  |  |
| *Pseudomonas syringae* |  |  | GCA_000012245.1 | GCA_000344335.2 |  |  |
| *Pseudomonas putida* |  |  | GCA_000007565.2 | GCA_000016865.1 |  |  |
| *Pseudomonas fluorescens* |  |  | GCA_000237065.1 | GCA_000009225.1 |  |  |
| *Pseudomonas amygdali* |  |  | GCA_002068135.1 | GCA_002905685.2 |  |  |
| *Pseudomonas Pyringae-group-gen* |  |  | GCA_000007805.1 | GCA_002966555.1 |  |  |
| *Pseudomonas stutzeri* |  |  | GCA_000590475.1 | GCA_000219605.1 |  |  |
| *Pseudomonas chlororaphis* |  |  | GCA_000698865.1 | GCA_000761195.1 |  |  |
| *Pseudomonas savastanoi* |  |  | GCA_000012205.1 | GCA_000164015.3 |  |  |
| *Pseudomonas mendocina* |  |  | GCA_000016565.1 | GCA_000204295.1 |  |  |
| *Pseudomonas fuscovaginae* |  |  | GCA_900108595.1 |  |  |  |
| *Pseudomonas oleovorans* |  |  | GCA_000297075.2 | GCA_000953455.1 |  |  |
| *Pseudomonas avellanae* |  |  | GCA_002905795.2 |  |  |  |
| *Pseudomonas mandelii* |  |  | GCA_000257545.3 |  |  |  |
| *Pseudomonas monteilii* |  |  | GCA_001534745.1 | GCA_000510285.1 |  |  |
| *Pseudomonas alcaligenes* |  |  | GCA_001597285.1 |  |  |  |
| *Pseudomonas fragi* |  |  | GCA_001543265.1 | GCA_002128325.1 |  |  |
| *Pseudomonas brassicacearum* |  |  | GCA_000194805.1 | GCA_000585995.1 |  |  |
| *Pseudomonas veronii* |  |  | GCA_002028325.1 | GCA_004919535.1 |  |  |
| *Pseudomonas-resinovorans* |  |  | GCA_000412695.1 |  |  |  |
| *Pseudomonas protegens* |  |  | GCA_000397205.1 | GCA_000012265.1 |  |  |
| *Pseudomonas-alcaliphila* |  |  | GCA_001941865.1 |  |  |  |
| *Pseudomonas synxantha* |  |  | GCA_000968415.2 | GCA_003851465.1 |  |  |
| *Pseudomonas fulva* |  |  | GCA_000213805.1 | GCA_002688705.1 |  |  |
| *Pseudomonas entomophila* |  |  | GCA_000026105.1 | GCA_003940785.1 |  |  |
| *Pseudomonas balearica* |  |  | GCA_000818015.1 |  |  |  |
| *Pseudomonas corrugata* |  |  | GCA_001708425.1 |  |  |  |
| *Pseudomonas cichorii* |  |  | GCA_000517305.1 |  |  |  |
| *Pseudomonas mosselii* |  |  | GCA_002309555.1 | GCA_002736065.1 |  |  |
| *Pseudomonas poae* |  |  | GCA_000336465.1 | GCA_004000515.1 |  |  |
| *Pseudomonas parafulva* |  |  | GCA_000800255.1 | GCA_002021815.1 |  |  |
| *Pseudomonas oryzihabitans* |  |  | GCA_001518815.1 |  |  |  |
| *Pseudomonas mucidolens* |  |  | GCA_900475945.1 |  |  |  |
| *Pseudomonas cremoricolorata* |  |  | GCA_000759535.1 |  |  |  |
| *Pseudomonas citronellolis* |  |  | GCA_001654435.1 | GCA_001586155.1 |  |  |
| *Pseudomonas azotoformans* |  |  | GCA_001579805.1 | GCA_002007785.1 |  |  |
| *Pseudomonas psychrotolerans* |  |  | GCA_001913135.1 |  |  |  |
| *Pseudomonas Soli* |  |  | GCA_000498975.2 |  |  |  |
| *Pseudomonas arsenicoxydans* |  |  | GCA_004135995.1 | GCA_900103875.1 |  |  |
| *Pseudomonas antarctica* |  |  | GCA_001647715.1 |  |  |  |
| *Pseudomonas koreensis* |  |  | GCA_001605965.1 | GCA_001654515.1 | GCA_003049825.1 |  |
| *Pseudomonas orientalis* |  |  | GCA_002934065.1 | GCA_003851585.1 | GCA_003851645.1 |  |
| *Pseudomonas libanensis* |  |  | GCA_003952245.1 |  |  |  |
| *Pseudomonas trivialis* |  |  | GCA_001186335.1 |  |  |  |
| *Pseudomonas taetrolens* |  |  | GCA_900475 | GCA_900637 |  |  |
| *Pseudomonas frederiksbergensis* |  |  | GCA_001874645.1 | GCA_001952935.1 | GCA_002355315.1 |  |
| *Pseudomonas lundensis* |  |  | GCA_001020725.2 |  |  |  |
| *Pseudomonas kribbensis* |  |  | GCA_003352185.1 |  |  |  |
| *Pseudomonas furukawaii* |  |  | GCA_002355475.1 |  |  |  |
| *Pseudomonas lurida* |  |  | GCA_002966835.1 |  |  |  |
| *Pseudomonas verPuta* |  |  | GCA_001294575.1 |  |  |  |
| *Pseudomonas yamanorum* |  |  | GCA_001612705.2 |  |  |  |
| *Pseudomonas ceraPi* |  |  | GCA_900074915.1 |  |  |  |
| *Pseudomonas alkylphenolica* |  |  | GCA_000746525.1 |  |  |  |
| *Pseudomonassp.ADP* |  |  | GCA_001465445.1 |  |  |  |
| *Brevundimonas diminuta* |  |  | GCA_004102925.1 |  |  |  |
| *Micavibrio aeruginosavorus* |  |  | *GCA_000226315.1* |  |  |  |
| *YerPinia enterocolitica* |  |  | GCA_000987925.1 |  |  |  |
| *Bacillus cereus* |  |  | GCA_006094295.1 |  |  |  |
| [*Staphylococcus*](C:/Users/wangchufang/AppData/Local/youdao/dict/Application/8.9.5.0/resultui/html/index.html#/javascript:;) [*aureus*](C:/Users/wangchufang/AppData/Local/youdao/dict/Application/8.9.5.0/resultui/html/index.html#/javascript:;) |  |  | GCA_000011265.1 | GCA_000017125.1 |  |  |
| [*Escherichia*](C:/Users/wangchufang/AppData/Local/youdao/dict/Application/8.9.5.0/resultui/html/index.html#/javascript:;) [*coli*](C:/Users/wangchufang/AppData/Local/youdao/dict/Application/8.9.5.0/resultui/html/index.html#/javascript:;) |  |  | GCA_003018455.1 |  |  |  |
| *Listeria monocytogenes* |  |  | GCA_900187225.1 |  |  |  |
| *salmonella enterica* |  |  | GCA_001558355.2 |  |  |  |
| *Campylobacter jejuni* |  |  | GCA_003999645.1 |  |  |  |
| *Cronobacter sakazakii* |  |  | GCA_000982825.1 |  |  |  |
| *Escherichia coli* |  |  | GCA_003697165.2 |  |  |  |
| *higella sonnei* |  |  | GCA_013374815.1 |  |  |  |
| *Vibrio parahaemolyticus* |  |  | GCA_001558495.2 |  |  |  |
| Total | 343 |  |  |  |  |  |

**Table S2.** Nucleic acid sequences of the targets specific for *P. aeruginosa* serogroup G

| **Genes** | ***Name of target Genes** | **Sequence length/bp** | **^#^Gene location** | **Encoded protein** | **Serotype specificity** |
| --- | --- | --- | --- | --- | --- |
| *wzzB* | *PA59_01887* | 1064 | 2003667- 2004731 | Chain length determinant protein | G (+) |
| *wbpA* | *PA59_01888* | 1269 | 2004841 -2006109 | UDP-N-acetyl-D-glucosamine 6-dehydrogenase | G (+) |
| *wbgU_1* | *PA59_01889* | 1023 | 2006150- 2007172 | UDP-N-acetylglucosamine 4-epimerase | G (+) |
| *group_234447* | *PA59_01891* | 1425 | 2007371 -2008795 | hypothetical protein | G (+) |
| *asnB_2* | *PA59_01893* | 1886 | 2009968 -2011851 | Asparagine synthetase [glutamine-hydrolyzing] 1 | G (-) |
| *e**psF_4* | *PA59_01894* | 1152 | 2011848- 2012999 | Putative glycosyltransferase EpsF | G (+) |
| *group_40682* | *PA59_01276* | 1579 | 1334921 -1336498 | hypothetical protein | G (+) |
| *asnB_1* | *PA59_05682* | 371 | 6043539- 6043907 | Asparagine synthetase [glutamine-hydrolyzing] 1 | G (-) |
| *group_71614* | *PA59_01892* | 1048 | 2008917 -2009963 | hypothetical protein | G (+) |
| *gnu* | *PA59_01896* | 822 | 2014314 -2015132 | N-acetyl-alpha-D-glucosaminyl-diphospho-ditrans,octacis-undecaprenol 4-epimerase | G (+) |
| *group_99977* | *PA59_00898* | 603 | 933619-934221 | hypothetical protein | G (-) |
| *group_234710* | *PA59_04268* | 915 | 4556294-4557208 | hypothetical protein | G (+) |
| *group_12136* | *PA59_01446* | 895 | 1527033 -1527926 | hypothetical protein | G (-) |
| *degU_1* | *PA59_01447* | 654 | 1528092-1528745 | Transcriptional regulatory protein DegU | G (-) |

*Reference strain is *P. aeruginosa PA 59*.

^#^The reference gene is GCA_009497675.1_ASM949767v1.

Result (+/-) indicate positive and negative signals.

**Table S3** . PCR detection limits for targets of *PA* serogroups G.

| Name of target genes | Primer set name | The detection limit of targets | |
| --- | --- | --- | --- |
|  |  | Pure *P. aeruginosa* (CFU/mL) | Artificial contamination (CFU/mL) |
| *group_40682* | G-PCR-1 | 4.0 ×10^3^ | 4.0 ×10^4^ |
| *wzzB* | G-PCR-4 | 4.0 ×10^3^ | 4.0 ×10^4^ |
| *wbpA* | G-PCR-5 | 4.0 ×10^3^ | 4.0 ×10^4^ |
| *wbgU_1* | G-PCR-6 | 4.0 ×10^3^ | 4.0 ×10^4^ |
| *group_234447* | G-PCR-7 | 4.0 ×10^3^ | 4.0 ×10^4^ |
| *epsF_4* | G-PCR-9 | 4.0 ×10^5^ | 4.0 ×10^6^ |
| *group_234710* | G-PCR-10 | 4.0 ×10^4^ | 4.0 ×10^5^ |
| *group_71614* | G-PCR-13 | 4.0 ×10^5^ | 4.0 ×10^6^ |
| *gnu* | G-PCR-14 | 4.0 ×10^3^ | 4.0 ×10^4^ |

**Table S4.** Real-time PCR results of specificity tests.

| Bacterial species | Strains ID | serogroup | Source* | Real -time PCR results |
| --- | --- | --- | --- | --- |
| *P. aeruginosa* | PA206052 | G | β | + |
|  | 16C02 | A | γ | - |
|  | 16C60 | B | γ | - |
|  | 16C38 | C | γ | - |
|  | 16C01 | D | γ | - |
|  | 17C44 | E | γ | - |
|  | 16C07 | F | γ | - |
|  | 206071 | H | β | - |
|  | 16C66 | I | γ | - |
|  | 17C70 | J | γ | - |
|  | 17C84 | K | γ | - |
|  | 16C58 | L | γ | - |
|  | 206070 | M | β | - |
| *Pseudomonas putida* | ^c^GIM 1.57 |  | α | - |
| *Pseudomonas fuscovaginae* | ST42-2 |  | α | - |
| *Pseudomonas pseudoalcaligenes* | ^a^CMCC1.1806 |  | α | - |
| *Pseudomonas mendocina* | ^a^CMCC1.1804 |  | α | - |
| *Pseudomonas fluorescens* | ^c^GIM1.492 |  | α | - |
| *Pseudomonas lini* | M41023-1 |  | α | - |
| *Pseudomonas mosselii* | ST42-10 |  | α | - |
| *Pseudomonas corrugata* | ST19-4 |  | α | - |
| *Pseudomonas oleovorans* | M43075-4 |  | α | - |
| *Pseudomonas taiwanensis* | 0617-3 |  | α | - |
| *Pseudomonas geniculata* | 52023-3 |  | α | - |
| *Staphylococcus aureus* | ^b^ATCC 22923 |  | α | - |
| *Escherichia coli* | ^b^ATCC 22922 |  | α | - |
| *Salmonella* | 837 |  | α | - |
| *Listeria monocytogenes* | 1333-2 |  | α | - |
| *Yersinia enterocolitica* | y2602 |  | α | - |

Result (±) indicate positive and negative signals.

* a CMCC, China Medical Culture Collection, China.

b ATCC, American Type Culture Collection, USA.

c GIM, Guangdong Institute of Microbiology, China.

d α, The Guangdong Institute of Microbiology, China; β, Guangdong Huankai Co., Ltd., China; γ, Zhujiang Hospital,Guangzhou,china.

Table S5 Number of novel targets identified change with the number of genomes/species analyzed

| Strain types | Increased number of whole genome sequences | Total number of whole genome sequences | Number of target genes of P.aeruginosa serogroup G | *P.aeruginosa* serogroup G target gene results obtained  at different whole genome numbers |
| --- | --- | --- | --- | --- |
| *P.aeruginosa* serogroup G strains |  | 62 | 5465 | 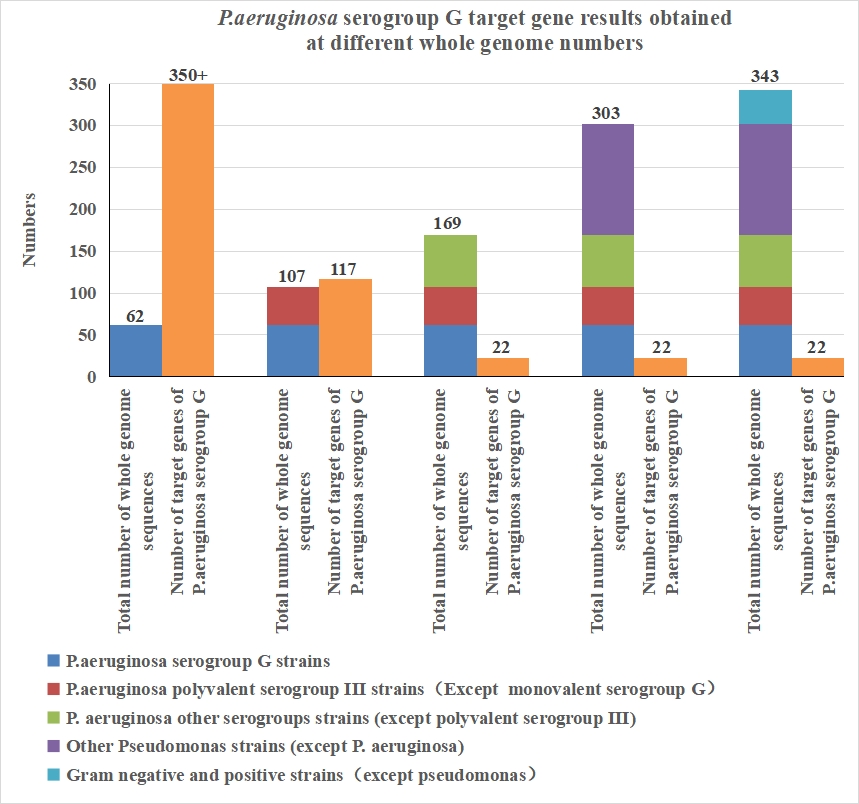 |
| *P.aeruginosa* polyvalent serogroup III strains | 45 | 107 | 117 |  |
| *P.aeruginosa* serogroups strains | 62 | 169 | 22 |  |
| *Pseudomonas spp.*strains | 137 | 302 | 22 |  |
| Gram negative and positive strains | 41 | 343 | 22 |  |

**
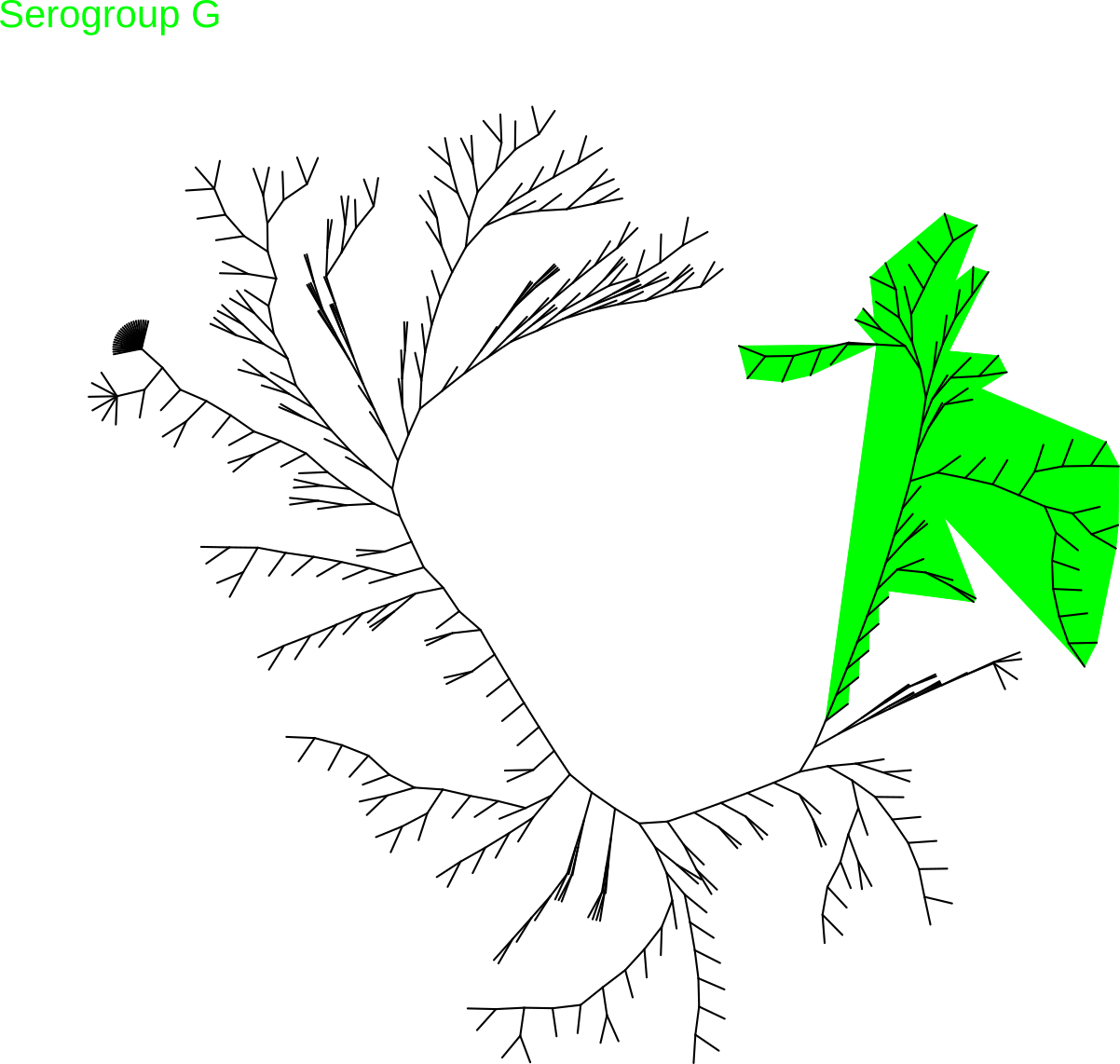
**

**Figure. S1. Phylogenetic anlysis of *P. aeruginosa* serogroup G.**

**S2.1**

**
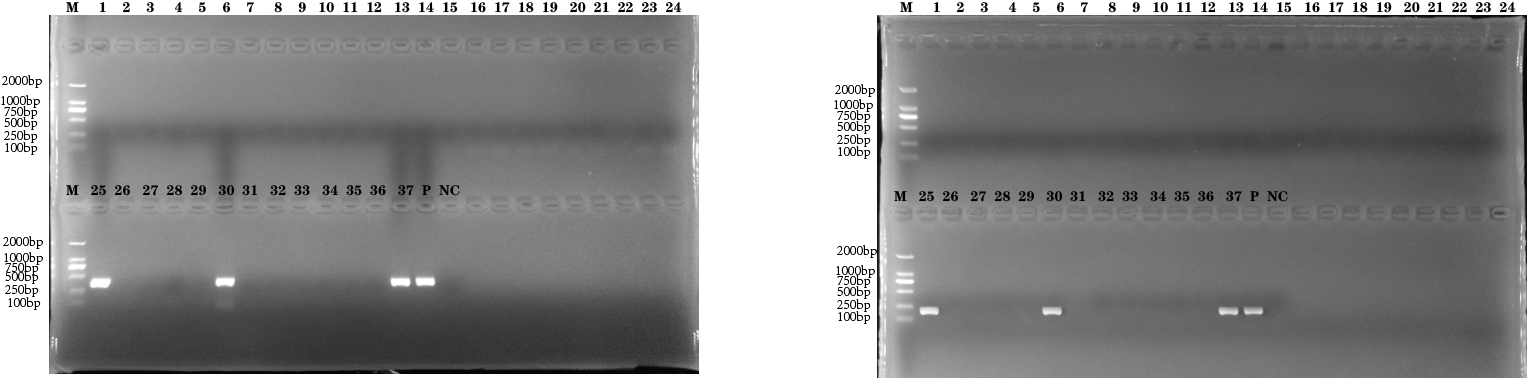
**

**S2.2**


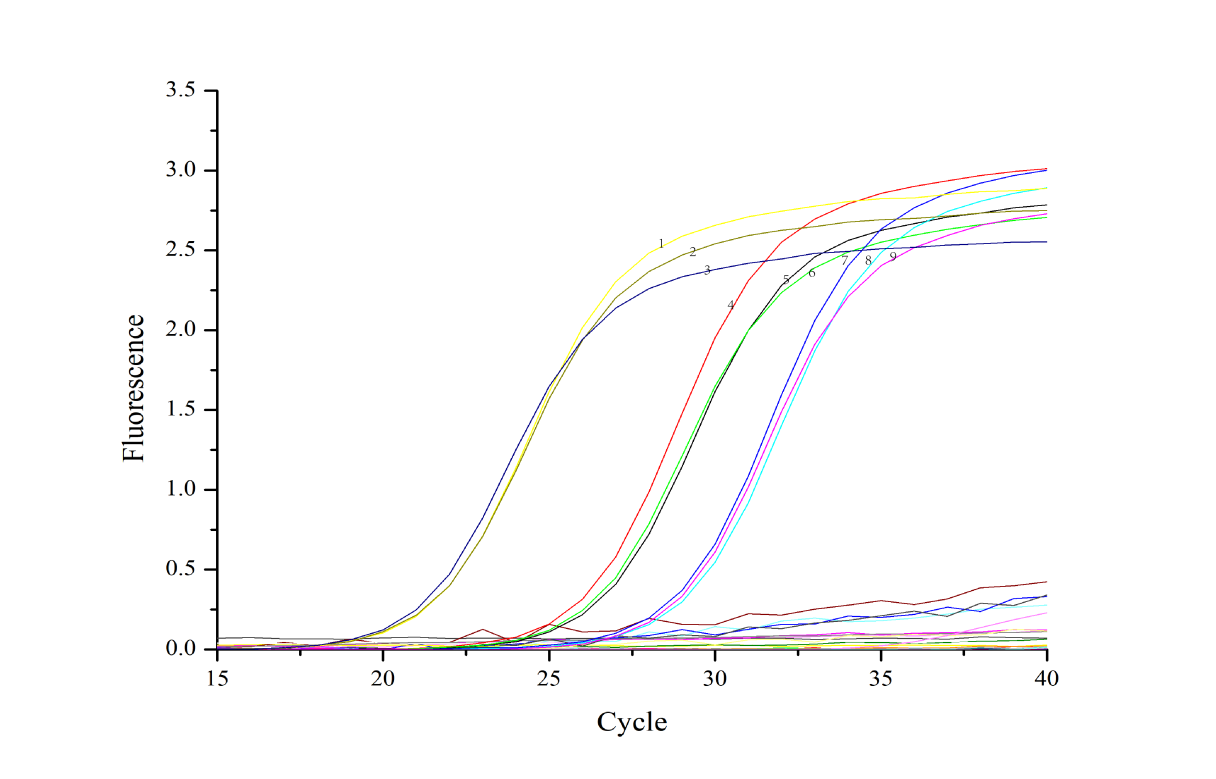


**Figure. S2.**Validation of the PCR method and real-time PCR for the detection of *P. aeruginosa* serogroup G in water samples. Lane M: DL DNA 2000 marker, lane NC: negative control, lane P: positive control, lanes 1–37 represent 37 water samples.

S2.1: Amplification using *PA59_01889* primers of PCR.

S2.2: Amplification using *PA59_01889* real-time PCR primers, curve1-3: three parallel samples of No.25; curve 4-6 three parallel samples of No.30; curve 7-9 three parallel samples of No.37.
